# Risk Factors and Etiology of Young Ischemic Stroke Patients in Estonia

**DOI:** 10.1155/2017/8075697

**Published:** 2017-06-18

**Authors:** Siim Schneider, Alina Kornejeva, Riina Vibo, Janika Kõrv

**Affiliations:** ^1^Department of Neurology and Neurosurgery, University of Tartu, Puusepa 8, 51014 Tartu, Estonia; ^2^Neurology Centre, North Estonia Medical Centre, Sütiste tee 19, 13419 Tallinn, Estonia; ^3^Faculty of Medicine, University of Tartu, Ravila 19, 50411 Tartu, Estonia

## Abstract

**Objectives:**

Reports on young patients with ischemic stroke from Eastern Europe have been scarce. This study aimed to assess risk factors and etiology of first-ever and recurrent stroke among young Estonian patients.

**Methods:**

We performed a retrospective study of consecutive ischemic stroke patients aged 18–54 years who were treated in our two hospitals from 2003 to 2012.

**Results:**

We identified 741 patients with first-ever stroke and 96 patients with recurrent stroke. Among first-time patients, men predominated in all age groups. The prevalence of well-documented risk factors in first-time stroke patients was 83% and in the recurrent group 91%. The most frequent risk factors were hypertension (53%), dyslipidemia (46%), and smoking (35%). Recurrent stroke patients had fewer less well-documented risk factors compared to first-time stroke patients (19.8 versus 30.0%, *P* = 0.036). Atrial fibrillation was the most common cause of cardioembolic strokes (48%) and large-artery atherosclerosis (LAA) was the cause in 8% among those aged <35 years. Compared to first-time strokes, recurrent ones were more frequently caused by LAA (14.3 versus 24.0%, *P* = 0.01) and less often by other definite etiology (8.5 versus 1.0%, *P* = 0.01).

**Conclusions:**

The prevalence of vascular risk factors among Estonian young stroke patients is high. Premature atherosclerosis is a cause in a substantial part of very young stroke patients.

## 1. Introduction

Knowledge of ischemic stroke in the young has changed considerably over the past decades. Its incidence in high-income countries has shown a rise among the young, whereas in the older age groups it has declined [1–5]. Stroke in the young was traditionally equated with rare causes and risk factors; however, this view has more recently been challenged. Accumulating evidence suggests that the prevalence of traditional risk factors in this patient age group is much larger than previously understood. These data mainly come from Western European and North American cohorts; however, reports on young patients from Eastern Europe have been lacking. The previous population-based registries from 1991–1993 and 2001–2003 found higher incidence of stroke in young Estonian patients compared to the Western-European countries. The results showed that Estonian men suffered stroke 2–7 years and women up to 5 years earlier than their Western-European counterparts [6, 7]. We hypothesized that this was primarily a consequence of the early accumulation of stroke risk factors. So we aimed to determine etiology and risk factor profiles in young Estonian stroke patients.

## 2. Methods

We set up a retrospective registry of consecutive patients aged 18–54 years who were treated in Tartu University Hospital and North Estonia Medical Centre, institutions with comprehensive stroke units and to where approximately two-thirds of stroke patients in Estonia are referred, from January 2003 to December 2012 with the discharge diagnosis of ischemic stroke (ICD-10 codes I63.0–I63.9). The cases were identified with the help of electronic discharge registry and all respective medical records were reviewed by the authors. Ischemic stroke was defined as a focal neurological deficit of acute onset lasting more than 24 hours or with evidence of acute brain ischemia on neuroimaging when symptoms last less than 24 hours. We excluded patients with transient ischemic attack, iatrogenic stroke, cerebral venous thrombosis, and hemorrhagic stroke.

All patients were clinically evaluated by a neurologist. The diagnostic workup was considered complete when all of the following were performed: brain imaging by computed tomography (CT) and/or magnetic resonance imaging (MRI); vascular imaging by ultrasonography, CT-angiography, magnetic resonance-angiography, and/or catheter angiography; and cardiac evaluation by echocardiography. If ECG revealed cardiac pathology, for example, atrial fibrillation, then further evaluation by echocardiography was not necessarily performed. ECG was performed on all patients, and 24-hour Holter ECG recording was done when considered clinically necessary. We classified stroke subtypes etiologically according to the Trial of Org 10172 in Acute Stroke Treatment (TOAST) criteria [8]. Cases of undetermined etiology were reviewed by two neurologists independently. Rare causes of stroke were considered as etiology of stroke only after complete investigation was negative for more common causes.

Ischemic stroke risk factors were registered and divided according to the American Heart Association/American Stroke Association guidelines for the primary prevention of stroke into well-documented and less well-documented groups. [9, 10]. The definition for hypertension was as follows: >140 mmHg systolic blood pressure and/or >90 mmHg diastolic blood pressure before stroke or 7 days after stroke or if on antihypertensive treatment. The criteria for dyslipidemia were serum total cholesterol ≥ 5.0 mmol/L, low-density lipoprotein cholesterol ≥ 3.0 mmol/L, high-density lipoprotein cholesterol < 1.0 mmol/L, or previous cholesterol-lowering therapy. Diabetes mellitus was diagnosed on the basis of one of the following criteria: fasting plasma glucose ≥ 7.0 mmol/L, two-hour postglucose challenge value ≥ 11.1 mmol/L, or glycated hemoglobin ≥ 6.5% or the patient was taking antidiabetic medication. Smoking, heavy drinking, and illicit drug use were listed as risk factors if they were mentioned in the medical records. Similarly, the patient was considered obese if indicated so in the medical records or if the body-mass index was ≥30 kg/m^2^. Infection was termed recent if any signs or symptoms occurred on admission or within one month prior to that. The diagnosis of patent foramen ovale (PFO) and atrial septal aneurysm (ASA) needed confirmation by transesophageal echocardiography (TEE).

Statistical analysis was performed using R [11]. Pearson chi-square test was used for comparing proportions; when expected counts were small, *P* values were computed by Monte Carlo simulation. Means were compared using the independent samples *t*-test. Values of *P* < 0.05 were considered statistically significant. Subgroup analysis was performed for sex and age groups of 18–44 and 45–54 years. For more detailed comparison with other studies TOAST subgroups were further divided according to age into groups of 18–34, 35–44, and 45–54 years. The Research Ethics Committee of the University of Tartu approved this study.

## 3. Results

We identified 1006 potential candidates in the hospital electronic database, of whom 837 fulfilled our inclusion criteria: 741 were first-ever strokes and 96 recurrent strokes. We excluded 169 patients for the following reasons: final diagnosis other than stroke, migraine, epilepsy, psychiatric disorder, cerebral venous thrombosis, and so forth (71); iatrogenic stroke (27); non-Estonian residents (5); and double registration in the database (66). Brain imaging with MRI was performed in 186 patients (22%), extra- and/or intracranial arteries were investigated in 626 patients (75%), and echocardiography was done in 587 patients (70%), of whom 129 patients (22%) were studied also with transesophageal echocardiography (TEE). Twenty-four-hour Holter ECG was recorded in 90 patients (11%).

### 3.1. First-Ever Stroke

**Figure 1:**
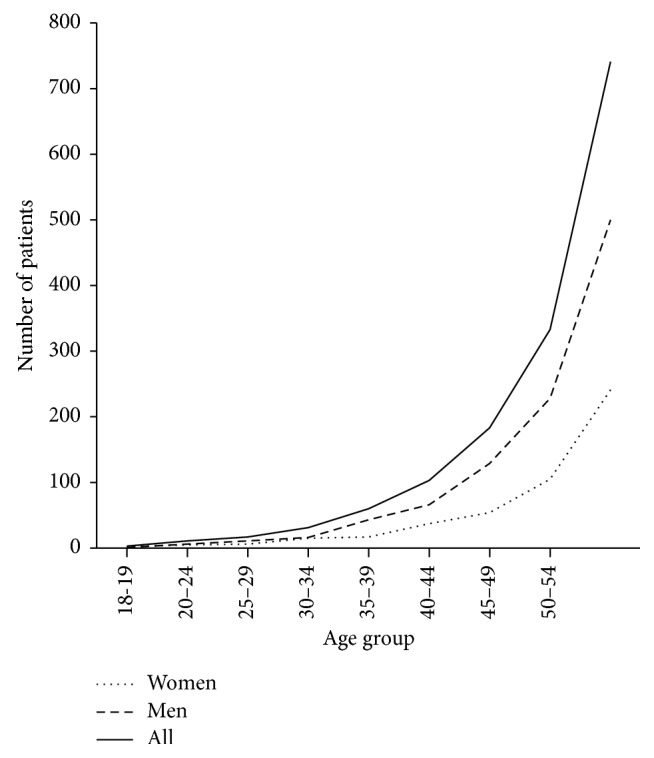
Patients with first-ever stroke according to age groups (number of men, women, and all).

Of the 741 first-ever stroke patients, 67.5% were men. Men predominated in all 5-year age bands (Figure 1). The age distribution between both sexes was equal.

**Figure 2:**
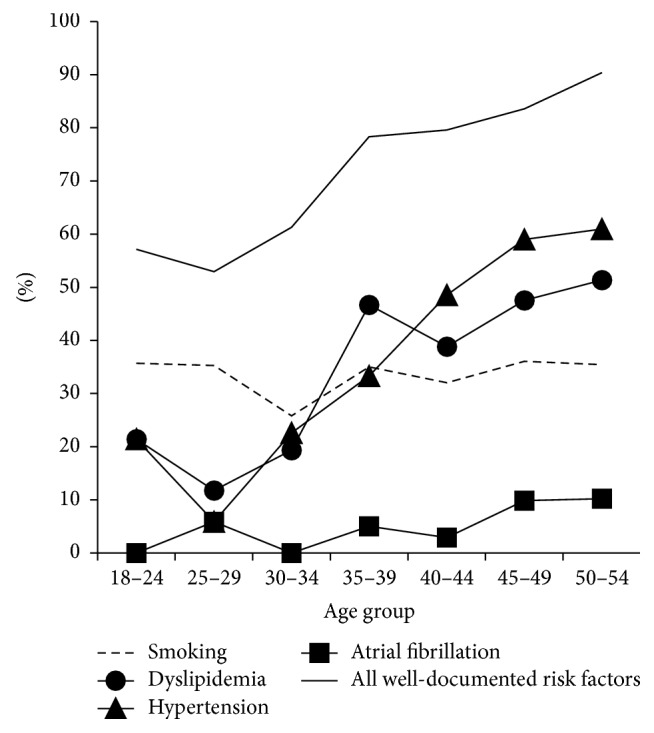
Prevalence of various vascular risk factors according to age in patients with first-ever stroke.

The prevalence of well-documented risk factors was 83.1% and it was significantly higher in men (87.2 versus 74.7%, *P* < 0.001) and in the older age group (88.0 versus 72.0%, *P* < 0.001). The most frequent risk factors were hypertension (52.9%), dyslipidemia (45.5%), and smoking (34.7%). Men more frequently had atrial fibrillation, coronary heart disease, and heart failure and were more often smokers (Table 1). Patients aged over 44 years suffered more often from dyslipidemia, hypertension, diabetes mellitus, coronary heart disease, and atrial fibrillation (Table 1, Figure 2).

While the overall prevalence of less well-documented risk factors did not show any sex disparity, women more often had migraine and recent infection, whereas men more frequently were heavy alcohol users. The prevalence of less well-documented risk factors was significantly lower in the older age group (40.4 versus 25.6%, resp., *P* < 0.001). The frequency of migraine, drug abuse, oral contraception, and gravidity or postpartum period was significantly lower in the older group (Table 1, Figure 2). In eighty-four patients (11.4%), among them 49 (9.8%) men and 35 (14.5%) women, no risk factors were identified.

**Figure 3:**
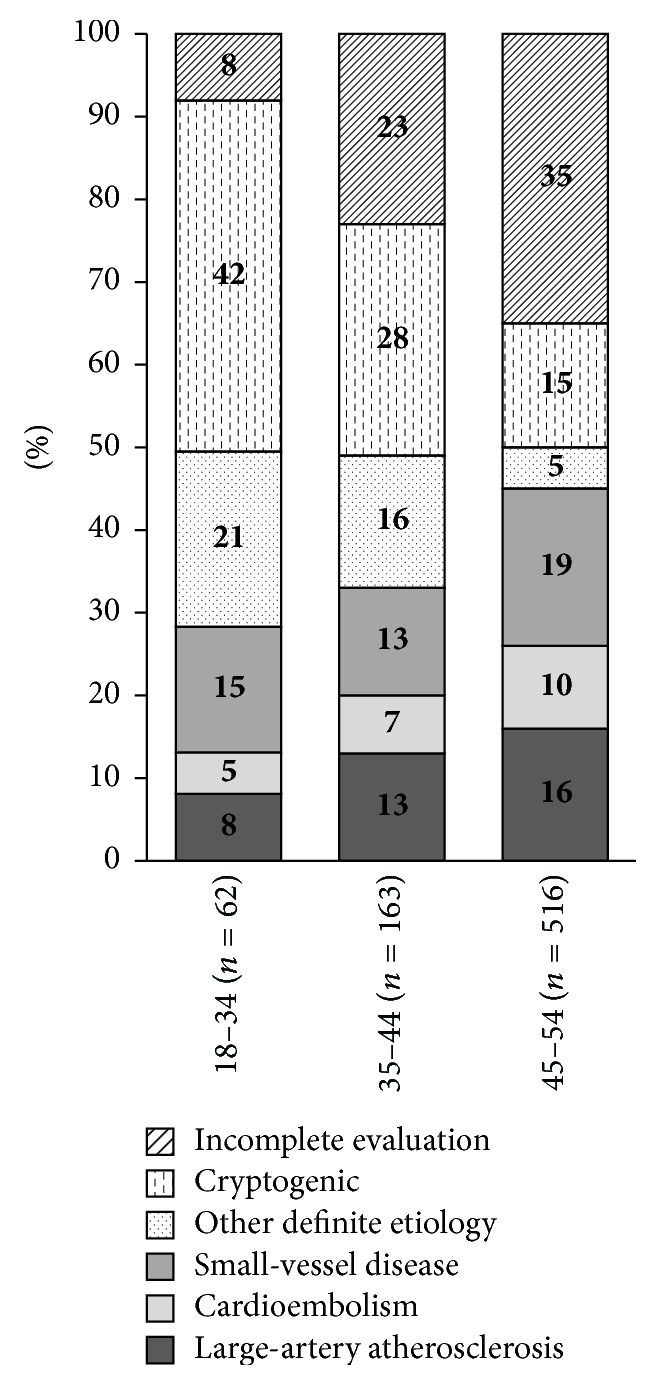
Frequency of etiologic subgroups in age groups of 18–34, 35–44, and 45–54 years. Cryptogenic stroke and incomplete evaluation comprise undetermined etiology according to TOAST.

Cardioembolism (CE, 17.1%) and large-artery atherosclerosis (LAA, 14.3%) were the most frequent known causes of ischemic stroke (Table 1, Figure 3). The causes of cardioembolism are shown in Table 2. CE and LAA were followed by small-vessel disease (SVD, 8.9%) and other definite etiology (ODE, 8.5%), the group in which cervical artery dissection was the leading cause of stroke (Table 3). Almost one in three patients had incomplete evaluation, 20.5% had negative evaluation despite extensive investigation (i.e., cryptogenic stroke), and 0.3% had two or more possible causes of stroke. Significant differences occurred in etiology between demographic groups (*P* < 0.001). Women had significantly more frequently ODE, while men tended to have LAA and CE as a cause of stroke with marginally missed significance. The proportion of ODE was significantly higher among younger patients (Table 1).

### 3.2. Recurrent Stroke

The proportion of men in the recurrent stroke group was 71.9%. Compared to patients with first-ever stroke, patients with recurrent stroke were older (46.9 versus 49.7 years, resp., *P* < 0.001) and had fewer less well-documented risk factors (30.0 versus 19.8%, resp., *P* = 0.036). The prevalence of well-documented risk factors was higher in the recurrent group, yet the significance was marginally missed (90.6 versus 83.1%, *P* = 0.060). The recurrent stroke patients more often had hypertension, diabetes mellitus, peripheral artery disease, and cardiac conditions other than atrial fibrillation, including acute myocardial infarction, cardiomyopathy, valvular heart disease, PFO and ASA, and cardiac tumors (Table 1). Five patients (5.2%), among them four (5.8%) men and one woman (3.7%), did not have any risk factors. Recurrent stroke was more frequently caused by LAA (14.3 versus 24%, resp., *P* = 0.01) and less often by ODE (8.5 versus 1.0%, resp., *P* = 0.01).

## 4. Discussion

We analyzed the risk factors and causes of ischemic stroke in a large, ethnically homogenous cohort of young patients hospitalized because of acute stroke between 2003 and 2012. Since data on young ischemic stroke patients from Eastern Europe are scarce, our study provides novel information on stroke characteristics in this population. As overall life expectancy and working age increase, it would be justified to widen the earlier age limit of 49 years for defining “young” stroke patients [12, 13]. Therefore, the upper age limit was 54 in our study, whereas in the Swiss [14] and international Fabry study [15] it was 55 years.

We report high prevalence of vascular risk factors, in both first-ever and recurrent stroke patients, 83% and 91%, respectively. The most common risk factors, namely, hypertension, dyslipidemia, and smoking, are in line with the previous three largest data sets of young ischemic stroke patients, the Helsinki Young Stroke Registry [12], 15 Cities Young Stroke Study [16], and Stroke in Young Fabry Patients (SIFAP) [17]. Our cohort, however, has notably higher prevalence of hypertension (53% versus 36–47%) and atrial fibrillation (8% versus 2–4%) and slightly higher rate of coronary heart disease (9% versus 4–6%), myocardial infarction (7% versus 3-4%), and heart failure (7% versus 1–5%). The higher frequency of well-documented risk factors in men and increasing age corroborates with the earlier studies [12, 17, 18]. The pooled data from 15 Cities study, FUTURE study, and SIFAP study [19] found a sharp rise in the prevalence of hypertension and dyslipidemia over age of 35. In our study the steep rise in the prevalence of hypertension, as well as the combined prevalence of all well-documented risk factors, started even earlier, at age of 30. Compared to the Estonian general population aged 18–54 years, the prevalence of hypertension and smoking was considerably higher in stroke patients (14% versus 53% and 28% versus 35%, resp.) [20].

To our knowledge the risk factor profile in recurrent young ischemic stroke patients has not been studied separately before. We speculate that the extremely high frequency of well-documented risk factors suggests that the secondary prevention had not been targeted sufficiently. Behavioral risk factors, namely, smoking, obesity, and heavy drinking, should presumably be significantly lower once the patient has survived first-time stroke, yet this assumption was not confirmed in our study. The proportion of patients without any stroke risk factors has varied from 5 to 27% across studies [12, 14, 17]; in our data it comprised 11% of first-time and 5% of recurrent patients.

We also found that men's predominance was the highest of previous reports [12, 14–16, 21] and surprisingly men prevailed in all age groups. In several European cohorts, women predominate among patients aged less than 30–35 years, that is, the most active reproductive age [12, 15, 16, 21], with the exception found by Naess et al. [22]. We suggest that men's predominance occurs due to the early heavy burden of well-documented risk factors that outweighs the women's sex-specific risk factors that usually prevail in this age group.

Our results regarding the overall proportion of LAA, CE, and SVD are similar to the earlier studies [12–15]. However, the relative age-specific proportions of TOAST subgroups reveal a higher rate of LAA (8 versus 0–6%) below age of 35 than previously reported [12, 13, 15, 23]. This very premature atherosclerosis could be the result of both early clustering of atherogenic risk factors and genetic susceptibility [24, 25].

Major differences existed also in the distribution of cardioembolic causes between our study and the other European cohorts [12, 13]. The rate of atrial fibrillation within CE group previously has been 14-15%, while in our patients it was 48%. It could be caused by a higher prevalence of hypertension in our cohort, which is the greatest attributable risk of atrial fibrillation [26]. The prevalence of other determined etiology was 8% and within it dissection comprised 38% in our study, both of which are lower than in most registries where the respective figures are roughly 25% and 50% [12–15, 23]. Our findings are at least partly attributable to the insufficient evaluation. The ODE group has low risk for recurrence [27, 28], and, as our data also confirm, their proportion in the recurrent stroke etiology is markedly smaller compared to the first-time events (1 versus 9%).

The definition of cryptogenic stroke (21% in our study) varies significantly across studies. We decided to classify low-risk cardioembolic causes as CE strokes and coagulopathies as ODE rather than cryptogenic stroke. Studies that have applied the same criteria have reported cryptogenic stroke ratios from 22% to 40% [12, 13]. As it is well recognized, the proportion of cryptogenic stroke decreased with age.

The limitations of our study are mostly derived from the retrospective and hospital-based design. However, data from the two biggest stroke centers incorporate most of the cases in our country. As a rule, all stroke patients in Estonia are admitted to the hospital, and state insurance covers the emergency medical care for all. Behavioral risk factors may be underreported, since they are difficult to extract retrospectively from medical records. Incomplete evaluation in about 30% of patients could mean that the currently high rate of large-artery atherosclerotic and cardioembolic strokes is probably even higher.

In conclusion, our unique findings, the greatest predominance of men, the highest prevalence of several well-documented risk factors, and the greatest rate of atherosclerosis under the age of 35 as a cause of stroke, raise the suspicion of the interaction of environmental and behavioral risk factor profile with the heritable component to stroke susceptibility. Our ongoing prospective registry of young stroke patients hopefully adds further knowledge to this current data.

## Figures and Tables

**Table 1:** Demographic data, risk factors, and etiology by sex and age groups.

	First-ever	Recurrent	*P*	Men	Women	*P*	Age 18–44	Age 45–54	*P*
	(*n* = 741)	(*n* = 96)		(*n* = 500)	(*n* = 241)		(*n* = 225)	(*n* = 516)	
Age, y	46.9 ± 7.4	49.7 ± 5.5	<0.001	47.2 ± 7.0	46.2 ± 8.2	0.083			
Men	500 (67.5)	69 (71.9)					143 (63.3)	357 (69.2)	0.133
							1.74^#^	2.25^#^	0.147
Well-documented risk factors	616 (83.1)	87 (90.6)	0.060	436 (87.2)	180 (74.7)	<0.001	162 (72.0)	454 (88.0)	<0.001
Hypertension	392 (52.9)	66 (68.8)	0.003	274 (54.8)	118 (49.0)	0.136	81 (36.0)	311 (60.3)	<0.001
Dyslipidemia	337 (45.5)	46 (47.9)	0.652	238 (47.6)	99 (41.1)	0.095	79 (35.1)	258 (50.0)	<0.001
Smoking	257 (34.7)	27 (28.1)	0.202	201 (40.2)	56 (23.2)	<0.001	73 (32.4)	184 (35.7)	0.398
Obesity	72 (9.7)	7 (7.3)	0.445	47 (9.4)	25 (10.4)	0.675	20 (8.9)	52 (10.1)	0.615
Diabetes mellitus	72 (9.7)	18 (18.8)	0.007	51 (10.2)	21 (8.7)	0.522	7 (3.1)	65 (12.6)	<0.001
Coronary heart disease	67 (9.0)	11 (11.5)	0.444	58 (11.6)	9 (3.7)	0.001	5 (2.2)	62 (12.0)	<0.001
Atrial fibrillation	59 (8.0)	10 (10.4)	0.411	51 (10.2)	8 (3.3)	0.001	7 (3.1)	52 (10.1)	0.001
Heart failure	49 (6.6)	10 (10.4)	0.171	40 (8.0)	9 (3.7)	0.029	12 (5.3)	37 (7.2)	0.355
Transitory ischemic attack	45 (6.1)	9 (9.4)	0.215	28 (5.6)	17 (7.1)	0.438	12 (5.3)	33 (6.4)	0.578
Other cardiac conditions	39 (5.3)	14 (14.6)	<0.001	31 (6.2)	8 (3.3)	0.100	11 (4.9)	28 (5.4)	0.763
Peripheral artery disease	8 (1.1)	5 (5.2)	0.011	8 (1.6)	0 (0.0)	0.059	1 (0.4)	7 (1.4)	0.447
Hormone replacement therapy	0 (0.0)	0 (0.0)	1.000						
Less well-documented risk factors	223 (30.1)	19 (19.8)	0.036	151 (30.2)	72 (29.9)	0.928	91 (40.4)	132 (25.6)	<0.001
Heavy drinking	130 (17.5)	13 (13.5)	0.327	111 (22.2)	19 (7.9)	<0.001	37 (16.4)	93 (18.0)	0.603
Migraine	36 (4.9)	0 (0.0)	0.027	14 (2.8)	22 (9.1)	<0.001	27 (12.0)	9 (1.7)	<0.001
Migraine with aura	23 (3.1)	0 (0.0)	0.097	9 (1.8)	14 (5.8)	0.003	15 (6.7)	8 (1.6)	<0.001
Recent or active infection	33 (4.5)	6 (6.2)	0.438	15 (3.0)	18 (7.5)	0.006	13 (5.8)	20 (3.9)	0.249
PFO	19 (2.6)	2 (2.1)	1.000	9 (1.8)	10 (4.1)	0.058	8 (3.6)	11 (2.1)	0.260
Oral contraception	13 (1.8)	0 (0.0)	0.382	NA	13 (5.4)		12 (14.6)	1 (0.6)	<0.001
Illicit drug use	6 (0.8)	0 (0.0)	0.624	5 (1.0)	1 (0.4)	0.670	5 (2.2)	1 (0.2)	0.011
Sleep apnea	6 (0.8)	0 (0.0)	0.624	6 (1.2)	0 (0.0)	0.185	2 (0.9)	4 (0.8)	1.000
Coagulopathy	4 (0.5)	0 (0.0)	1.000	3 (0.6)	1 (0.4)	1.000	1 (0.4)	3 (0.6)	1.000
Pregnancy or postpartum period	3 (0.4)	0 (0.0)	1.000	NA	3 (1.2)		3 (3.7)	0 (0.0)	0.038
Stroke subtypes			<0.001			<0.001			<0.001
LAA	106 (14.3)	23 (24.0)	0.014^*∗*^	82 (16.4)	24 (10.0)	0.019^*∗*^	26 (11.6)	80 (15.5)	0.158^*∗*^
Small-vessel disease	66 (8.9)	9 (9.4)	0.880^*∗*^	40 (8.0)	26 (10.8)	0.212^*∗*^	14 (6.2)	52 (10.1)	0.090^*∗*^
Cardioembolism	127 (17.1)	19 (19.8)	0.519^*∗*^	98 (19.6)	29 (12.0)	0.011^*∗*^	30 (13.3)	97 (18.8)	0.070^*∗*^
ODE	63 (8.5)	1 (1.0)	0.009^*∗*^	32 (6.4)	31 (12.9)	0.003^*∗*^	39 (17.3)	24 (4.7)	<0.001^*∗*^
Undetermined etiology	379 (51.1)	44 (45.8)	0.551^*∗*^	248 (49.6)	131 (54.4)	0.225^*∗*^	116 (51.6)	263 (51.0)	0.883^*∗*^
Undetermined etiology (subgroup)			0.248			0.010			<0.001
Two or more causes	2 (0.5)	1 (2.2)		2 (0.8)	0 (0.0)		0 (0.0)	2 (0.4)	
Negative evaluation	152 (40.1)	14 (32.6)		87 (35.1)	65 (49.6)		72 (32.0)	80 (15.5)	
Incomplete evaluation	225 (59.4)	29 (65.2)		159 (64.1)	66 (50.4)		44 (19.6)	181 (35.1)	

Data are expressed as mean SD or *n* (%); ASA, atrial septal aneurysm; LAA, large artery atherosclerosis; ODE, other definite etiology; PFO, patent foramen ovale; TOAST, Trial of Org 10172 in Acute Stroke Treatment; Cardiac conditions other than atrial fibrillation include acute myocardial infarction, cardiomyopathy, valvular heart disease, PFO and ASA, and cardiac tumors. ^#^Men/women. ^*∗*^Post hoc test; values of *P* < 0.01 are statistically significant (Bonferroni correction).

**Table 2:** Sources of cardioembolism in first-ever stroke patients.

	*n* = 127	%
*High-risk sources*		
Atrial fibrillation	61	48%
Recent myocardial infarction	12	9%
Cardiomyopathy	7	6%
Endocarditis	7	6%
Sick sinus syndrome* *	5	4%
Intracardiac thrombus	5	4%
Mechanical heart valve* *	4	3%
Rheumatic valve disease	4	3%
Congestive heart failure	3	2%
Ventricular wall akinesia	2	2%
PFO + ASA	2	2%
Myxoma* *	1	1%
Congenital cardiac malformation	1	1%
*Sources of low or uncertain risk*		
PFO	7	6%
Hypokinetic left ventricular segment	4	3%
ASA	1	1%

**Table 3:** Subgroups of other determined etiology.

	*n* = 63	%
Dissection	25	40%
Hematologic disease	10	16%
Active malignancy	7	11%
Vasculitis	5	8%
Migrainous infarction	5	8%
Illicit drug use	3	5%
Pregnancy and puerperium related	3	5%
Vascular malformation/aneurysm	2	3%
Factor V Leiden mutation	1	2%
Protein C deficiency	1	2%
Coarctation of aorta	1	2%
